# The treatment of chemotherapy-induced peripheral neuropathy: a review of current management options and a potential role for scrambler therapy

**DOI:** 10.3389/fpain.2025.1607102

**Published:** 2025-07-07

**Authors:** Hassan Aboumerhi, Henry Vucetic, Andrew Gruenzel, Bahar Moftakhar, Mona Gupta, Santosh K. Rao, Michael D. Staudt

**Affiliations:** ^1^ Department of Anesthesiology and Perioperative Medicine, Division of Pain Medicine, University Hospitals Cleveland Medical Center, Cleveland, OH, United States; ^2^ Department of Hematology and Oncology, University Hospitals Cleveland Medical Center, Seidman Cancer Center, Cleveland, OH, United States; ^3^ Case Western Reserve University, School of Medicine, Cleveland, OH, United States; ^4^ Department of Hospice and Palliative Medicine, University Hospitals Cleveland Medical Center, Cleveland, OH, United States; ^5^ Department of Neurological Surgery, University Hospitals Cleveland Medical Center, Cleveland, OH, United States

**Keywords:** cancer, chemotherapy-induced peripheral neuropathy, chronic pain, neuropathic pain, neuropathy, review, scrambler therapy

## Abstract

Chemotherapy-induced peripheral neuropathy (CIPN) presents a growing medical and financial burden on patients and the healthcare system alike. This has been treated with conservative and interventional care limited by efficacy, side effects, and lack of coverage. As such, there is an unmet treatment need for effective non-invasive or minimally invasive therapies for the treatment of CIPN. Scrambler therapy (ST) is a peripheral, non-invasive neuromodulation technique, which uses transcutaneous electrical stimulation to modulate pain signals. ST has shown mixed results in clinical trials; while some patients report symptom relief, more robust evidence is required before it can be widely recommended. This review article outlines the burden of CIPN and the current state of treatment, including pharmacological and interventional therapies. The emerging data on ST and its role in treating CIPN is highlighted, including a review of published observational and randomized controlled trials. We also discuss the gaps and challenges ahead in establishing this therapy as a standard of care.

## Introduction

Chemotherapy-induced peripheral neuropathy (CIPN) is a dose-limiting side effect of several cancer chemotherapeutic agents that profoundly impacts quality of life and survivorship (1). Symptoms may arise from a single high-dose exposure or from cumulative effects of chemotherapy, often necessitating dose adjustments or early treatment discontinuation, which can worsen oncologic outcomes (2). The incidence depends on the drug, dose, combinations, duration, pre-existing diseases such as diabetes or chronic kidney disease, and individual susceptibility. Higher incidence rates have been seen with platinum-based compounds, vinca alkaloids, taxanes, and proteasome inhibitors with platinum agents being most neurotoxic (3). The incidence of CIPN is approximately 50%–90%. More than 80% of cancer survivors develop acute CIPN, which decreases patient tolerance to treatment (4). The incidence of CIPN approaches nearly 100% for some agents at higher doses, although differences in the definition, evaluation and reporting can lead to large variability in the reported occurrence (5). The prevalence of CIPN ranges from 19% to more than 85% (6).

CIPN primarily affects the peripheral nervous system, leading to sensory, motor, and autonomic dysfunction. The pathology differs based on the type of chemotherapy used. Platinum-based agents cause axonopathy, with preferential damage to large, myelinated sensory fibers in the dorsal root ganglia (5). This leads to stocking-glove sensory loss and neuropathic pain. Taxanes lead to microtubule dysfunction, impairing axonal transport, leading to distal sensory loss and pain (7). Bortezomib, a proteasome inhibitor, induces mitochondrial and endoplasmic reticulum stress, resulting in painful, distal sensory neuropathy (8).

In addition to the detrimental effects on pain and function, there is a significant economic burden associated with the development of CIPN. Patients with CIPN have significantly higher utilization of healthcare resources, including an increased chance of hospitalization, increased visits to the emergency department or outpatient clinic, and higher medication utilization (9). One database study of privately insured administrative claims records found that patients with CIPN on average had $17,344 (USD) higher healthcare costs during a 12-month period compared to patients without CIPN (10). In addition to higher direct costs related to treatment, indirect costs are also higher due to patient and caregiver work loss and disability costs (11). For example, pain, paresthesias and weakness in hands and/or feet secondary may lead to impaired dexterity, trouble feeling or manipulating objects, and difficulties ambulating—all of these factors may negatively impact a patient's ability to return to work or perform work-related tasks (12, 13).

## The current state of management for chemotherapy-induced peripheral neuropathy

The standard of care for managing CIPN encompasses both preventive strategies and therapeutic interventions. No pharmacologic agents have demonstrated consistent efficacy in preventing CIPN. The American Society of Clinical Oncology (ASCO) advises against the use of several agents for CIPN prevention due to insufficient evidence, including acetyl-L-carnitine, amitriptyline, gabapentin/pregabalin, and calcium-magnesium (14).

ASCO advises clinicians to evaluate and discuss with patients the potential benefits of delaying, reducing, or discontinuing chemotherapy or switching to alternative agents, when patients experience severe CIPN (15). ASCO recommends duloxetine as the primary pharmacologic treatment for established CIPN. Clinical trials have shown that duloxetine can reduce neuropathic pain associated with CIPN (14). As a serotonin-norepinephrine reuptake inhibitor, duloxetine enhances the availability of the key neurotransmitters involved in activating the descending pain-modulating pathway (16). It may also reduce inflammation and nerve injury by inhibiting the activation of p38 and NF-kB (17). While tricyclic antidepressants and anticonvulsants are used for neuropathic pain, their efficacy in treating CIPN remains unproven. ASCO does not recommend these agents for CIPN treatment due to limited supporting evidence (14).

Several complementary and alternative medicines have been tested for treating CIPN. Exercise, acupuncture, mindfulness practices, yoga, meditation, and touch therapies like acupressure, reflexology, and massage, have been found to reduce CIPN symptoms and improve quality of life (4). Engaging in regular physical activity including stretching, walking, resistance training, and balance exercises, may help alleviate CIPN symptoms and improve functional outcomes (18). In addition, some nutrients and herbal medicines have shown potential therapeutic effects in patients with neuropathic pain (19).

The use of intrathecal pain therapies has a storied history in the treatment of refractory cancer pain (20), and both morphine and ziconotide are FDA-approved agents. The pivotal studies which formed the basis for FDA-approval reported on the treatment of refractory cancer pain, with most patients having mixed neuropathic and nociceptive pain (21, 22). It has been suggested that cancer patients with a life expectancy of 3–6 months or greater are appropriate candidates; however, its use has generally fallen out of favor due to changing practice patterns, as well as the need for frequent pump refills and potential device-related complications. Furthermore, there is no literature on the use of intrathecal therapy in the treatment of CIPN specifically, with most studies and current practice being focused on nociceptive or mixed pain.

There is significant enthusiasm for the use of spinal cord stimulation (SCS) in the treatment of CIPN. Preclinical studies have been supportive of its use in attenuating pain, such as demonstrating the inhibition of gait impairment and paclitaxel-induced mechanical and cold hypersensitivity in rats (23, 24). There are numerous case reports and case series supporting the use of SCS and dorsal root ganglion stimulation in the treatment of CIPN and other painful polyneuropathies (25, 26); however, its use is currently considered off-label. This is in contrast to the treatment of painful diabetic neuropathy, which is supported by level I evidence and is FDA-approved (27). Altogether, this suggests that SCS is a viable and potentially effective treatment option for CIPN, although more robust data are needed.

CIPN is both an acute and chronic complication of chemotherapy, with many patients developing lasting and debilitating pain (28). The current standard of care for treatment relies on a multimodal approach primarily consisting of medications, although side effect profiles, tolerance and dependency remain significant challenges. Although implantable neuromodulation devices have demonstrated efficacy, there is no robust data for either intrathecal therapy or SCS, and furthermore patients may not want to undergo a surgical procedure for pain control. As such, there is an unmet treatment need for non-invasive or minimally invasive therapies for the treatment of CIPN.

## Scrambler therapy: mechanisms and indications

**Figure 1:**
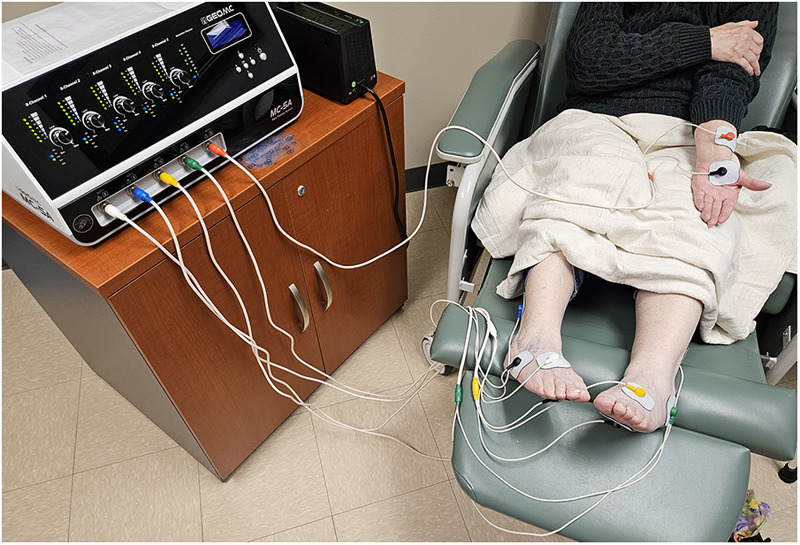
Example of the hardware and patient setup for scrambler therapy. Electrodes are applied to the painful areas to be treated.

Scrambler therapy (ST), an electro-analgesia therapy, is a non-invasive treatment for chronic neuropathic and cancer-related pain (Figure 1). ST uses an algorithm to generate painless stimulation programs, which are then transmitted to the central nervous system transcutaneously (29, 30). The principle behind ST lies in its ability to disrupt the transmission of pain signals by replacing them with artificial pain-free signals (31). The mechanism of action of ST is not fully understood, but several theories have been proposed. The inventor of ST believes it works by stimulating C fiber surface receptors, which are responsible for transmitting sensory information, including pain, to the brain (29, 30). By delivering electrical stimulation through these receptors, ST may be able to “override” the pain signals, effectively blocking their transmission and reducing the patient's perception of pain. Another theory suggests that ST induces neuroplastic changes in the brain, essentially retraining the nervous system to interpret the signals from the affected area as non-painful through a series of repeated treatments (32). It is believed that ST alters pain perception at the brain level to relieve pain (33).

ST is typically administered in a series of daily sessions, lasting approximately 30–60 min. Electrodes are placed on the skin at the pain site and along corresponding dermatomes. A low-intensity electrical current is carefully calibrated by the operator to ensure appropriate non-painful stimulation. ST has demonstrated effectiveness in alleviating various types of neuropathic pain, including post-herpetic neuralgia, diabetic neuropathy, and complex regional pain syndrome (34). Furthermore, ST has been explored as a potential treatment for cancer-related pain, specifically CIPN (29, 30).

The indications for ST continue to evolve, but it is generally considered an option for patients with chronic neuropathic and cancer-related pain. Appropriate patient selection is critical for successful outcomes, as individuals with certain underlying conditions or psychological factors may be less likely to respond to the treatment. ST does seem very promising for the treatment of difficult types and patterns of pain, although it is not a cure for chronic pain. Some patients may experience a recurrence of pain after a period, requiring additional treatment sessions to maintain the benefits (35).

Theoretically, there is risk of seizure with lead placement across the head, especially in patients with pre-existing seizure disorders. There may be risk of arrhythmia if placed across the heart, particularly if patients have a history of arrhythmia. But no such cases of seizures or arrhythmia have been reported in the literature. Leads should not be placed across or near implanted stimulation devices such as pacemakers, defibrillators, and SCSs. If necessary, the provider should consider placing these devices in surgical mode and arranging appropriate device management as needed should leads need to be placed in close proximity. ST initiation should be carefully set to avoid painful overstimulation, especially in allodynic or hypersensitive patients. A meta-analysis reported rare cases of contact dermatitis and minor ecchymosis at lead placement sites (47). ST safety has not been evaluated in patients who are pregnant or nursing.

## The role of scrambler therapy in chemotherapy-induced peripheral neuropathy

ST is being studied and utilized as a means of reducing CIPN symptoms in the extremities, such as burning pain, dysesthesias, numbness, tingling, and motor dysfunction. By reducing these symptoms, patients can ideally lead more normal lives with less pain, improved sleep, and better function of their extremities. ST is primarily performed on patients who have completed chemotherapy but continue to have CIPN symptoms for months-to-years following treatment. While protocols vary, most institutions utilizing this therapy for CIPN offer 10 treatment sessions over the course of 2 weeks and will subsequently monitor for efficacy and duration of relief (36). If symptoms do improve significantly, but recur later, additional treatments may be considered at the discretion of the provider.

ST represents a novel, non-invasive, low-risk treatment option for those with lasting neuropathic symptoms after completion of their cancer treatment. Despite FDA-approval in 2009, Medicare and most commercial payers do not reimburse ST. Most patients pay a cash price per session or series of sessions. There are a small, but growing number of pain centers in the United States that offer ST. Many patients self-refer while others are recommended by an increasing number of specialists, particularly oncologists managing chemotherapy. Having more effective treatment options may allow some patients to maintain their chemotherapeutic regimen with less concern for debilitating neuropathic pain afterwards. Currently there is no research evaluating the neuroprotective effect on patients who are actively undergoing chemotherapy, but this could be a direction for future study.

## Current evidence for scrambler therapy

The role of ST in treating CIPN has been investigated in a limited number of pilot and randomized controlled trials, which are summarized in Table 1.

**Table 1:** Summary of clinical trials for the use of ST in the treatment of CIPN.

Study	Study Design	N	Eligibility	Intervention	Comparison	Results
Smith et al., (37)	Observational	16	Adults, pain score > 5	10 consecutive 60 min sessions of ST	None	Reduction in pain score of 59% by the end of ten days. No change in quality of life.
Campbell et al., (38)	Randomized double-blind	14	Adults, CIPN > 6 months, pain score ≥ 4/10	10 consecutive 50 min sessions of ST	Active sham device delivering a just perceptible electrical sensation, non-therapeutic	No significant difference in pain score between ST and sham.
Coyne at al., (39)	Observational	39 (33 with CIPN)	Adults, pain score > 5 or numbness that bothered them “at least a little bit”	10 consecutive 45 min sessions of ST	None	Reduction of pain score of 35% at 14-day follow-up. Persistent improvement in pain at 3 months. Persistent improvement in sensory and motor scores at 3 months. Improvements in quality of life.
Pachman et al., (40)	Observational	37	Adults, CIPN ≥ 1 month, tingling or pain ≥ 4/10	10 consecutive 30 min sessions of ST	None	At ten days, average pain decreased by 53%; average tingling decreased by 44%; average numbness decreased by 37%
Tomasello et al., (41)	Observational	9	Pediatric, pain score >5, unresponsive to 1–4 pain medications	10 consecutive 45 min sessions of ST	None	Dramatic improvement in pain score, activity impairment, mood, ambulation, sleep, and interpersonal relationships
Smith et al., (42)	Randomized sham-controlled	35	Adults, CIPN ≥ 3 months, pain ≥ 4/10	10 consecutive 30 min sessions of ST	10 consecutive 30 min sessions of “sham” electrode placement	No difference in pain, sensory or motor score between arms.
Loprinzi et al., (15)	Randomized non-sham-controlled	50	Adults, CIPN ≥ 3 months, tingling or pain ≥ 4/10	10 consecutive 30 min sessions of ST	Home TENS use for 14 consecutive 30 min sessions	Decrease in pain and tingling symptoms to a moderate degree, more than TENS % of patients with ≥50% reduction in symptoms after 2 weeks
Childs et al., (43)	Randomized non-sham-controlled	50, cross over analysis from Loprinzi et al.	Adults, CIPN ≥ 3 months, tingling or pain ≥ 4/10	10 consecutive 30 min sessions of ST	Home TENS use for 14 consecutive 30 min sessions	In crossover phase, 60% (6 of 10) ST treated patients, and 25% (3 of 12) TENS treated patients reported ≥50% reduction in symptoms.
Chung et al., (44)	Observational	10	Adults, CIPN ≥ 3 months	10 consecutive 45 min sessions of ST	None	Improved quality of life measures at 6 months: Improved pain, pressure & cold tolerance, walking, numbness, sleep. Decreased medication use. 82% high-satisfaction rate

The first published trial evaluating the use of ST for the treatment of CIPN was published in 2010 (37). In this pilot study, 16 patients with CIPN received 10 daily one-hour ST treatments (37). There was a reduction in pain score by 59% by the end of treatment course. Despite these significant findings, most patients reported that their pain returned to pre-treatment levels 1 or 2 months after the end of therapy. There was also no change in quality-of-life metrics.

Two additional studies were published in 2013. Campbell et al. reported the first randomized double blind trial comparing ST to a novel active sham device designed to deliver a just perceptible electrical sensation (38). 14 patients were randomized to receive one of the two treatments for 10 daily 50 min sessions. There was no significant difference in the primary endpoint of change in pain between the treatment arms. Conversely, Coyne et al. found improvement in pain score at 14 days, 1 month, 2 month and 3 months of follow-up in an observational study (39). They also noted improvements in the sensory and motor components of CIPN, as well as improvement in general activity, mood, sleep and enjoyment of life.

In another study, 37 patients with CIPN received consecutive daily 30 min ST sessions for 10 days, after which their average pain score decreased by 52%. These patients' tingling score reduced by 44% and numbness score by 37%. Patients also reported improved quality of life over the course of treatment and at ten weeks follow-up (40).

Another pilot study randomized patients to ST or sham placement of electrodes. There was no significant difference in pain between the ST group and the sham group at 10, 28, 60 or 90 days, with small non-significant improvement in pain in both arms. Both arms demonstrated improvement in sensory and motor scores, suggesting the sham electrode placement may have had some therapeutic effect (42).

Loprinzi et al. compared ST to trans-electrical nerve stimulation (TENS) with 50 patients randomized to receive either two weeks of ST or two weeks of TENS therapy (15). A greater percentage of patients of receiving ST reported at least 50% reduction in pain scores and tingling scores compared to patients who received TENS (56% vs. 28% and 48% vs. 24%, respectively). More patients receiving ST were more likely to recommend the treatment they received than patients receiving TENS. This cohort of patients was further evaluated in a crossover analysis, where 22 of the 50 patients proceeded to the crossover phase. Patients were observed for 8 weeks then were allowed to cross over for an additional 10 weeks (2 weeks of treatment and 8 weeks of observation). The same trend was noted during the cross over phase of this trial, with 60% of the ST patients reaching ≥50% reduction in symptoms compared to only 25% of the TENS treated patients (43).

A pilot study showed ST may have a meaningful impact on quality-of-life measures impaired by CIPN. Patients were followed for 6 months post-ST. In addition to decreased pain, they displayed improved pressure and cold tolerance and improved ambulation. Patients exhibited decreased numbness and tingling and improved sleep. 82% of patients expressed high satisfaction and all denied adverse events (44).

Little is known about ST in pediatric patients. A study on 9 adolescents between the ages of 12 and 17 showed promise in treating pediatric CIPN. ST resulted in dramatic improvements in pain score, activity impairment, mood, ambulation, sleep, and interpersonal relationships (41).

There are many recurrent limitations across these trials, including low enrollment, short follow-up, and lack of a control arm. These studies have demonstrated mixed results, emphasizing the need for further research to understand the role of ST in the management of CIPN.

## The future of scrambler therapy

CIPN can only be expected to become more prevalent as the population ages, cancer rates increase, and more patients receive chemotherapy. Patients with CIPN require solutions beyond medications with high side effect profiles. There are ongoing studies worldwide investigating wearable therapies, cryotherapy, and novel medications. ST has proven potential and is likely to play a growing role in the future of CIPN treatment. Compared to many medications, it does not carry a significant systemic side effect profile. Compared to implantable therapies such as SCS or intrathecal therapy, ST carries less potential risk as a transcutaneous treatment as well as lower overall cost. Collaboration with relevant specialists will be key to improving access to this therapy. Oncologists, neurologists, pain specialists, and physiotherapists should familiarize themselves with ST to provide the right resources for patients.

A challenge of ST therapy is the lack of standardization. Studies comparing session length such as 30 or 60 min may provide more uniform care and results. Likewise, investigations could clarify if less or more than 10 sessions are necessary for a clinically significant result. These results will contribute to a more standardized, replicable therapy (45). One of the inconveniences of ST is the time commitment required. Identifying the most effective number and length of sessions may expand access of this therapy. Future studies may show less and/or shorter sessions are required for clinically meaningful relief, but that evidence does not exist at this time. Successful, large-scale randomized control trials may help achieve Medicare and commercial payer coverage for ST. Currently, there is no evidence suggesting males or females are more responsive to ST. Further study in the pediatric population is necessary as well.

ST has potential in treating certain aspects of CIPN. Studies could further investigate its potential efficacy in treating numbness compared to pain compared to motor symptoms. Studies could be tailored towards specific chemotherapeutic agents, which could better prognosticate ST efficacy (46). ST should be evaluated for its role in treating CIPN during chemotherapy for acute pain relief, or potentially as a prophylactic therapy for the prevention of CIPN.

The time investment on behalf of the patient and possible need for repeat therapy leave questions on dosing and durability that remain unanswered. Nevertheless, ST is associated with minimal side effects and invasiveness, and its promising efficacy makes it an attractive and growing therapy, even if it is unlikely to be standalone. As studies continue, ST will likely serve as an adjunct or part of a multi-modal approach to treating CIPN. These nuances will have to be tailored to the individual patient as more data continues to emerge.

## Conclusion

ST offers a low-risk option for patients who have already known significant risk from their cancer diagnosis through chemotherapy and resultant CIPN. Many patients are tired of expensive medications with variable efficacy and high side effect profiles. Others are not interested in more invasive procedures after the ordeal of cancer treatment. ST is non-invasive and shows significant promise in treating CIPN. As studies continue, treatments should become more standardized, as should our ability to better prognosticate success and durability.
